# Influences of TPU Content on the Weld Line Characteristics of PP and ABS Blends

**DOI:** 10.3390/polym15102321

**Published:** 2023-05-16

**Authors:** Thanh Trung Do, Van-Thuc Nguyen, Huynh Do Song Toan, Pham Son Minh, Tran Minh The Uyen, Trung H. Huynh, Vinh Tien Nguyen, Van Thanh Tien Nguyen

**Affiliations:** 1 Ho Chi Minh City University of Technology and Education, Ho Chi Minh City 71307, Vietnam; 2 Department of Industrial Engineering and Management, National Kaohsiung University of Science and Technology, Kaohsiung 80778, Taiwan; 3 Faculty of Mechanical Engineering, Industrial University of Ho Chi Minh City, Nguyen Van Bao Street, Ward 4, Go Vap District, Ho Chi Minh City 70000, Vietnam

**Keywords:** compatible, injection molding, tensile test, fracture surface, Taguchi method

## Abstract

This study aims to explore the effects of Thermoplastic Polyurethane (TPU) content on the weld line properties of Polypropylene (PP) and Acrylonitrile Butadiene Styrene (ABS) blends. In PP/TPU blends, increasing the TPU content results in a significant decrease in the PP/TPU composite’s ultimate tensile strength (UTS) and elongation values. Blends with 10 wt%, 15 wt%, and 20 wt% TPU and pure PP outperform blends with 10 wt%, 15 wt%, and 20 wt% TPU and recycled PP in terms of UTS value. The blend with 10 wt% TPU and pure PP achieves the highest UTS value of 21.85 MPa. However, the blend’s elongation decreases due to the poor bonding in the weld line area. According to Taguchi’s analysis, the TPU factor has a more significant overall influence on the mechanical properties of PP/TPU blends than the recycled PP factor. Scanning electron microscope (SEM) results show that the TPU area has a dimple shape on the fracture surface due to its significantly higher elongation value. The 15 wt% TPU sample achieves the highest UTS value of 35.7 MPa in ABS/TPU blends, which is considerably higher than other cases, indicating good compatibility between ABS and TPU. The sample containing 20 wt% TPU has the lowest UTS value of 21.2 MPa. Furthermore, the elongation-changing pattern corresponds to the UTS value. Interestingly, SEM results present that the fracture surface of this blend is flatter than the PP/TPU blend due to a higher compatibility rate. The 30 wt% TPU sample has a higher rate of dimple area than the 10 wt% TPU sample. Moreover, ABS/TPU blends gain a higher UTS value than PP/TPU blends. Increasing the TPU ratio mainly reduces the elastic modulus of both ABS/TPU blends and PP/TPU blends. This study reveals the advantages and disadvantages of mixing TPU with PP or ABS to ensure that it meets the requirements of the intended applications.

## 1. Introduction

Polymers have become necessary because of their excellent mechanical properties, lightweight, low-temperature processing, low cost, and recycling ability [1,2,3]. Since the invention of synthetic polymers more than a century ago, the polymer has evolved to play an important role. Polymers, for example, could be combined with other materials to form new composite materials. Authors have recently discovered advanced polymers with unique properties, such as conductive polymers, bio-medical polymers, and shape memory polymers [4,5,6]. Polymer products can be manufactured using various techniques, including injection molding, extrusion molding, blowing, stamping, and additive manufacturing [7,8,9,10,11].

**Figure 1:**
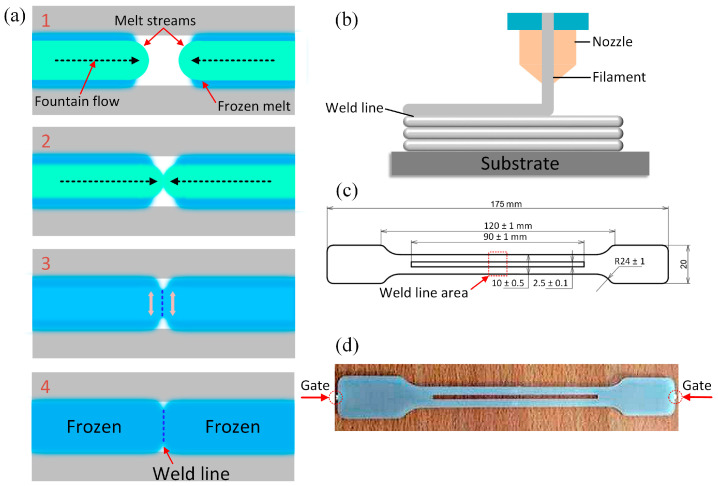
Weld line mechanism and test sample: (**a**) weld line mechanism in injection molding, (**b**) weld line mechanism in 3D printing, (**c**) testing sample under the standard of ASTM D638, (**d**) injection sample.

The weld line is a critical feature of the final product in the injection molding process. The weld line formation occurs when two molten plastic streams merge or when a molten plastic stream contacts a solid one, as illustrated in Figure 1a,b. Figure 1a shows the formation of weld line during the injection molding process, which includes 4 steps: cooling, meeting, weld line nucleation, and weld line formation. The weld line in an injection molding product can significantly impact the strength and durability of the last part [12,13,14,15]. This weld line can act as a weak spot in the material, reducing the part’s strength and durability [16,17]. By minimizing the impact of the weld line on the part’s strength and durability, manufacturers can produce high-quality injection-molded products that meet the needs of their customers. For example, Kagitci et al. [18] optimized injection molding parameters such as injection pressure, injection time, and packing pressure to reduce the impact of the weld line on the quality of glass fiber-reinforced polyamide. Leong et al. [19] performed film-inserted molding before injection. They demonstrated that the interaction between the film and the weld line could improve mechanical properties by distorting the weld line orientation [20]. Additionally, according to Barad et al. [21], reducing the channel thickness can decrease the distance of the weld line’s influence on the ultimate tensile strength (UTS). Additionally, it may be necessary to use specialized equipment, such as twin-screw extruders or mixing screws, to ensure proper mixing and dispersion of the composite materials or some unique molding process as mold pre-heating [22], gas-assisted injection molding [23], over-molding [24], or thin wall injection molding [25]. Overall, the injection molding process for composite materials requires careful attention to processing parameters, material selection, and equipment to ensure that the resulting parts meet the desired specifications for strength, durability, and appearance.

Polypropylene (PP) and acrylonitrile butadiene styrene (ABS) are thermoplastic polymers with high strength, ductility, toughness, and chemical resistance. Because of the high adhesive capacity of TPU, PP and ABS can be mixed with it to improve the bonding of the blends, particularly in the weld line area. According to De León et al. [26], mixing 10–30 wt% TPU and ABS improves layer adhesion due to hydrogen bonding between molecules. As a result, the strength between layers and the platform is increased. Luo et al. [27] generated TPU/PP blends using a traditional twin-screw extruder and a non-twin-screw extruder technique. Compared to the conventional twin-screw extruder method, the non-twin-screw extruder offers better interaction and dispersion between TPU and PP. Additionally, it was demonstrated by Kannan et al. [28] that adding nano clay to TPU/PP blends enhanced the dispersion between TPU and PP. He et al. [29] created PP film capacitors by coating an aluminum layer. Kuo et al. [30] examined the weld strength of samples produced by friction welding from polylactic acid (PLA) and PC (polycarbonate) polymers. Li et al. [31] revealed that the fiber-reinforced polymer could be repaired effectively by using 3D-printed patches. At the same time, Zhu et al. [32] reported that the longitudinal critical refraction wave could measure the residual welding stress. Interestingly, Chen et al. [33] proved that the hydrogel generated by a room temperature reaction followed by a freeze-thaw and annealing treatment demonstrated the best overall mechanical characteristics. However, despite playing a significant role in the mechanical properties of the blended product, the weld line characteristics of these PP/TPU and ABS/TPU blends are rarely investigated. Adding TPU can increase the overall cost of the injection molding process, and it may reduce the stiffness and melt strength of the material blend.

In this report, we aim to survey the effects of TPU on the weld line characteristics of PP/TPU and ABS/TPU blends. The ratios of TPU and recycled PP of the blends are carefully examined. The blend samples are conducted with the tensile test. After that, the fracture surface is observed via a scanning electron microscope. This study indicates the advantages and disadvantages of mixing TPU with PP or ABS. It tests the material under realistic conditions to ensure that it meets the requirements of the intended application.

## 2. Experimental Methods

**Figure 2:**
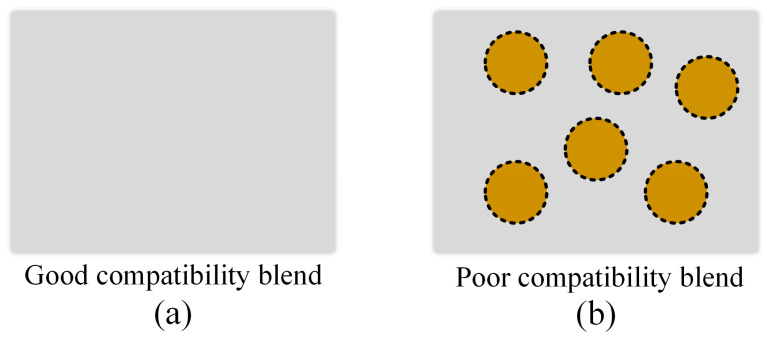
Blending compatibility mechanism: (**a**) good compatibility blend, and (**b**) poor compatibility blend.

The ABS 750 SW polymer used in this investigation is produced by Kumho Petrochemical in Korea with MFR/MVR and Vicat softening points of 38 g/10 min and 95 °C, respectively. Dongguan Rayan Polymer in China produces the TPU polymer (TPU^®^ TU90AE), which has a Vicat softening point of 85.5 °C and MFR/MVR of 79 g/10 min. Advanced Petrochemical Company in Saudi Arabia produces the PP polymer (Advanced-PP 1100 N), whose MFR/MVR and Vicat softening points are 12 g/10 min and 154 °C. The injection molding machine type is Haitian-MA 1200III. The molding conditions for the testing samples are presented in Table 1. For each case, we injected four samples; therefore, the average mechanical properties have standard deviation values or error bars. Before injection molding, the initial PP, ABS, and TPU samples are dried. TPU percentages range from 10 wt% to 30 wt%. In the PP/TPU blend, besides pure PP, recycled PP is also mixed, as shown in Table 2. The PP/TPU blend and ABS/TPU blend are prepared following ASTM D638 shape, as shown in Figure 1c, with the grips distance of 135 mm. The injection samples are tested by a tensile test machine AG-X Plus 20 kN (Shimadzu, Japan) at a 5 mm.min^−1^ speed. Then, the fracture surfaces are evaluated by a scanning electron microscope (SEM) TM4000 (Hitachi, Japan) [29,30]. The schematic of the blending compatibility mechanism is shown in Figure 2.

## 3. Results and Discussion

### 3.1. PP/TPU Blends

In this section, we present the effects of TPU percentage on PP/TPU blend properties. In addition, the impacts of recycled PP on the PP/TPU blend are also mentioned. Mixing with more than 40 wt% TPU could generate voids, leading to poor adhesion between the TPU and PP matrix [31]. Therefore, the TPU ratio ranges from 10 wt% to 30 wt%, while recycled PP varies from 0 wt% to 100 wt%, as shown in Table 1. The experiment samples are divided into five groups. Different groups have different ratios of pure PP and recycled PP. While in each group, various cases have different TPU percentages. After testing with the same injection condition, the UTS value of the pure PP is 25.2 MPa, while the UTS value of recycled PP is 22.3 MPa. The recycled PP is recycled over one time. Compared to pure PP, the degradation of recycled PP is the reason for reducing the mechanical properties.

**Figure 3:**
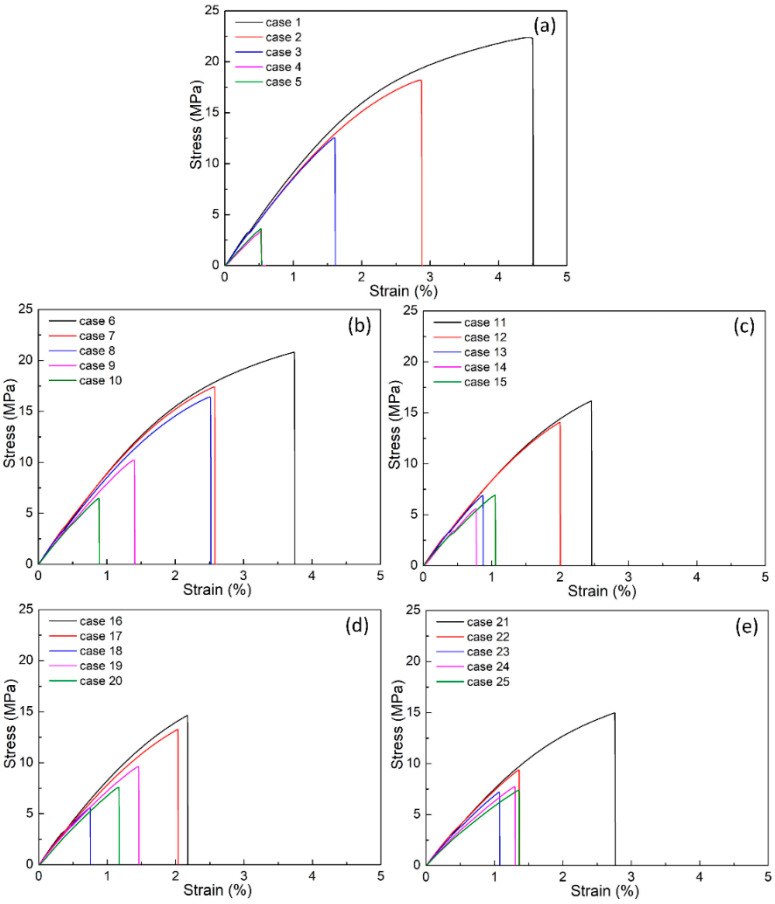
Stress-strain diagrams of PP/TPU samples at different groups: (**a**) group 1, (**b**) group 2, (**c**) group 3, (**d**) group 4, and (**e**) group 5.

Figure 3 presents the stress-strain curves of the PP/TPU blend samples with different TPU, pure PP, and recycled PP compositions. The findings show that adding more TPU polymer in each group primarily causes a decrease in the PP/TPU composite’s ultimate tensile strength (UTS), a consistent result of Lin et al.’s [32] report. The reason for the reduction of the UTS value is the low strength of TPU. At the same time, in these cases, the poor bonding in the weld line area results in a drop in the ductility of the blend. The weld line reduces the continuity of the sample structure as it forms from two cooled streams in opposite directions, as shown in Figure 1a,b [13]. Furthermore, the mechanical properties of the PP/TPU blend vary between groups, and these differences are investigated in the following figures.

**Figure 4:**
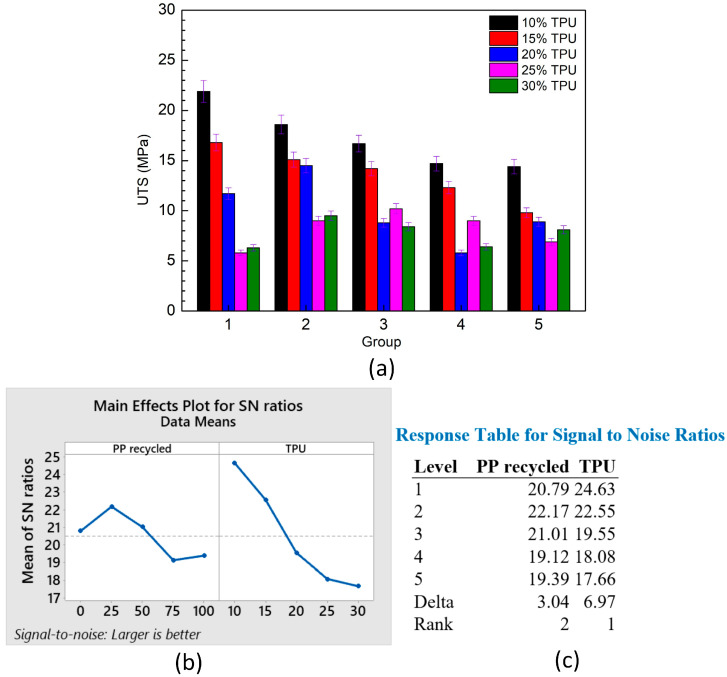
Average tensile strength of PP/TPU samples at different TPU and PP percentages: (**a**) UTS—group graphs, (**b**) main effects plot for means, and (**c**) response table for means.

Figure 4a shows the average tensile strength of PP/TPU samples at different TPU and PP percentages. Blending with 10–20 wt% TPU and pure PP (group 1) gains a higher UTS value than blending recycled PP (group 5). The highest UTS value of 21.9 ± 1. 2 MPa is achieved by a blend of 10 wt% TPU and 90 wt% pure PP. In contrast, a blend of 90 wt% recycled PP and 10 wt% TPU reaches a UTS value of 14.4 ± 0.8 MPa. The reason is the higher strength of pure PP compared to recycled PP. However, when the TPU percentage surpasses 20 wt%, blends with recycled PP have a higher UTS value. This phenomenon could be explained by the excellent mixture between the recycled PP and the TPU molecule when the TPU percentage is high enough. The lower average molecular weight of the recycled PP facilitates the blending process with the TPU molecule.

In group 2, the pure PP rate is between 52.5–67.5 wt%, higher than the recycled PP rate, 17.5–22.5 wt%. On the contrary, in group 4, the recycled PP rate is 52.5–67.5 wt%, higher than the pure PP rate, 17.5–22.5 wt%. When comparing groups 2 and 4 with the same TPU percentage, samples with a high pure PP rate have a higher UTS than samples with a high recycled PP rate. In group 3, when the percentage of pure and recycled PP is the same, increasing TPU also results in a decline in the UTS value. When comparing five groups in samples with 10–15 wt% TPU, increasing TPU reduces the UTS value of the PP/TPU blends in all pure and recycled PP cases. Moreover, in models with 20–30 wt% TPU, mixing with 25–50 wt% recycled PP (group 2, 3) results in higher UTS values than in other cases. As previously stated, this phenomenon is due to the excellent mixture of recycled PP and TPU molecules.

Moreover, the results are analyzed via the Taguchi method. The analysis applies Minitab software with L25 orthogonal array, 2 factors, and 5 levels. Factor PP recycled has 0 wt%, 25 wt%, 50 wt%, 75 wt%, and 100 wt%. Factor TPU has levels of 10 wt%, 15 wt%, 20 wt%, 25 wt%, and 30 wt%. Figure 4b,c shows the main effects plot for means of the UTS value with the “larger is better” target and the response table for means. The TPU percentage rank is 1, while the percentage of recycled PP is 2. Therefore, the TPU factor impacts are more substantial than the recycled PP factor. The regression equation of the UTS value is:

UTS = 23.04 − 0.0378 *×* PP recycled − 0.4900 *×* TPU (1)

This equation indicates the negative effects of recycled PP and TPU on the UTS values of the blends.

**Figure 5:**
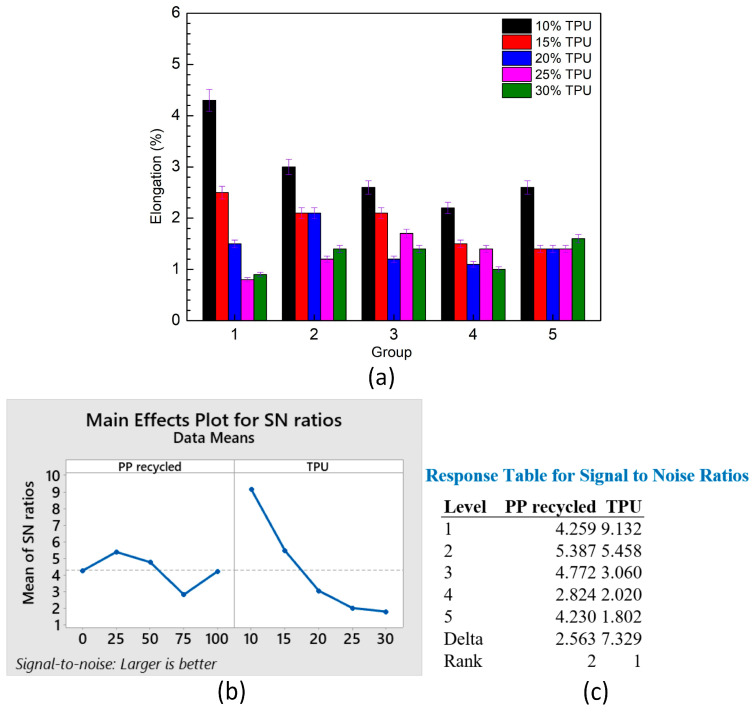
Average elongation of PP/TPU samples at different TPU and PP percentages: (**a**) elongation group graphs, (**b**) main effects plot for means, and (**c**) response table for means.

Figure 5a shows the average elongation of PP/TPU samples at different TPU and PP percentages. The elongation values range from 0.8 ± 0.06% to 4.3 ± 0.15%. The low elongation values of these samples relate to the weld line in the injection samples, and a weld line reduces the continuity of the sample structure [13]. In addition, similar to the changing pattern in the UTS value, samples with 10–15 wt% TPU primarily reduce the elongation values when increasing the recycled PP percentage. In samples with 20–30 wt% TPU, mixing with 25–50 wt% recycled PP (group 2, 3) mostly leads to higher elongation values than other cases. In other words, increasing the UTS value also improves the ductility of the PP/TPU blends. Moreover, due to the low compatibility with the PP matrix, the elongation is reduced when mixing TPU with the PP matrix. Compared to pure PP, the degradation of recycled PP is the reason for reducing the mechanical properties, presenting the negative impacts of the recycled PP content factor.

Figure 5b,c show the main effects plot for means of the elongation value with the “larger is better” target and the response table for means. The TPU percentage rank is 1, while the percentage of recycled PP is 2. The TPU factor impacts are stronger than the recycled PP factor. The regression equation of the elongation value is presented as follows:

Elongation = 3.600 − 0.00464 × PP recycled − 0.0796 × TPU(2)

This equation presents the negative effects of recycled PP and TPU on the elongation values of the blends.

**Figure 6:**
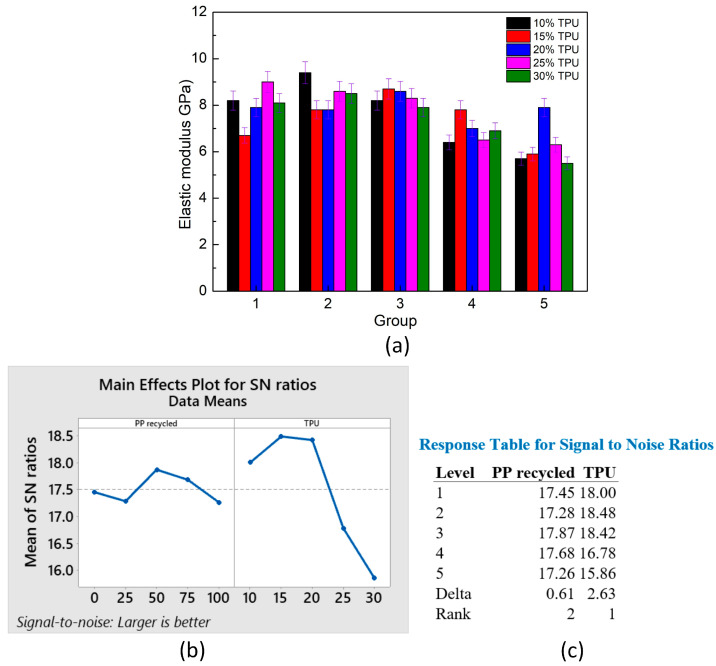
Average elastic modulus of PP/TPU samples at different TPU and PP percentages: (**a**) elastic modulus group graphs, (**b**) main effects plot for means, and (**c**) response table for means.

Figure 6 shows the average elastic modulus of PP/TPU samples at different TPU and PP percentages. Young’s modulus values range from 5.5 ± 0.4 GPa to 9.4 ± 0.6 GPa. Considering the pure and recycled PP, the average Young’s modulus values vary from 7.4 ± 0.6 GPa to 7.8 ± 0.6 GPa. This result indicates that the factor of pure and recycled PP slightly impacts the elastic modulus value. However, considering that the TPU ratio strongly affects the elastic modulus value, samples with 10–20 wt% TPU gain a high average elastic modulus, ranging from 8.0 GPa to 8.4 GPa.

On the contrary, samples with 25–30 wt% TPU suffer a decline in the average elastic modulus, ranging from 6.3 GPa to 6.9 GPa. This issue is because when the TPU ratio is high enough, it conglomerates into clusters rather than scattering on the PP matrix. Therefore, due to the ductility of TPU, increasing the TPU ratio mainly leads to a decline in the elastic modulus. Moreover, because of the low compatibility between TPU and the PP matrix, the elastic modulus declined when mixing TPU with the PP matrix. Compared to pure PP, the degradation of recycled PP is the reason for reducing the elastic modulus, presenting the negative impacts of the recycled PP content factor.

Figure 6b,c show the main effects plot for the means of the elastic modulus value with the “larger is better” target and the response table for means. The TPU percentage rank is 1, while the percentage of recycled PP is 2. The TPU factor impacts are more substantial than the recycled PP factor. The regression equation of the elastic modulus value is presented as follows:

Elastic modulus = 9.568 − 0.00016 *×* PP recycled − 0.0988 *×* TPU (3)

This equation shows the negative effects of recycled PP and TPU on the elastic modulus values of the blends.

**Figure 7:**
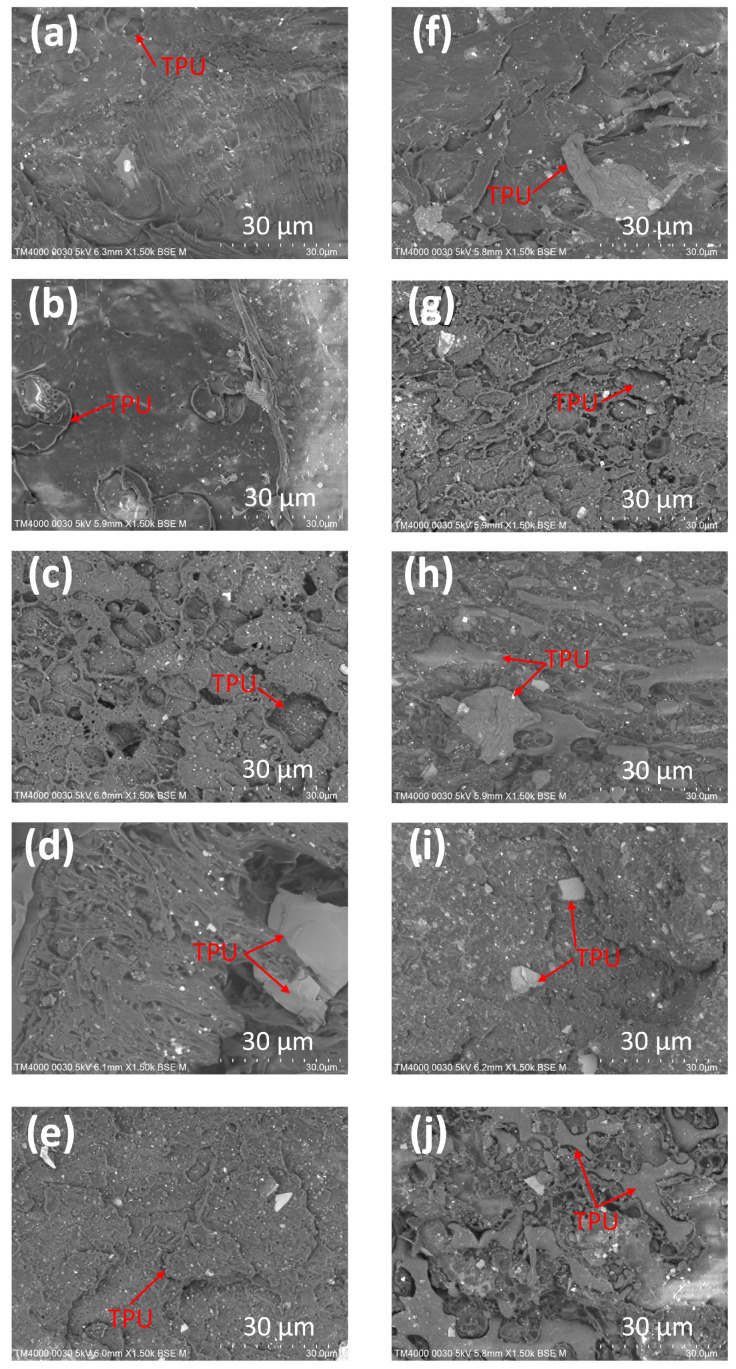
SEM picture of the fracture surface of samples with pure PP and recycled PP: (**a**–**e**) group 1 corresponds to cases 1–5, and (**f**–**j**) group 2 corresponds to cases 6–10.

Two groups with and without recycled PP are selected to observe the fracture surface’s microstructure. Figure 7 shows the scanning electron microscopy of the PP/TPU blends in a group with TPU and pure PP and another with TPU, pure PP, and recycled PP. Figure 7a–e represent samples in group 1 with 10 wt%, 15 wt%, 20 wt%, 25 wt%, and 30 wt% TPU cases mixed with pure PP. Similarly, Figure 7f–j correspond to samples in group 2 containing 10 wt%, 15 wt%, 20 wt%, 25 wt%, and 30 wt% TPU cases mixed with both pure and recycled PP. Some white dots in the figure could present CaCO_3_ particles because adding calcium carbonate to PP could improve processing efficiency, mechanical properties, and thermal properties while reducing the cost. Because PP and TPU are distinct phases, the boundary between them is obvious. From 10 wt% to 20 wt% TPU, the blending is mixed well. However, from 20 wt% to 30 wt% TPU, there are some TPU islands due to the low compatibility with the PP matrix. The TPU area has a dimple shape on the fracture surface due to its high elongation. Without TPU, PP has a smooth fractured surface [23]. Therefore, the sample surface becomes rougher when increasing the TPU ratio. When comparing samples with and without recycled PP, the samples with recycled PP have a rougher fracture surface. This phenomenon indicates a lower ductility, consistent with the stress-strain curve diagrams. Remarkably, the rough surface and the separation of PP and TPU phases suggest a poor compatibility mechanism between them, as shown in Figure 2b.

### 3.2. ABS/TPU Blends

This section examines the effects of the TPU ratio on the ABS/TPU blends. ABS/TPU blends are prepared with 10–30 wt% TPU.

**Figure 8:**
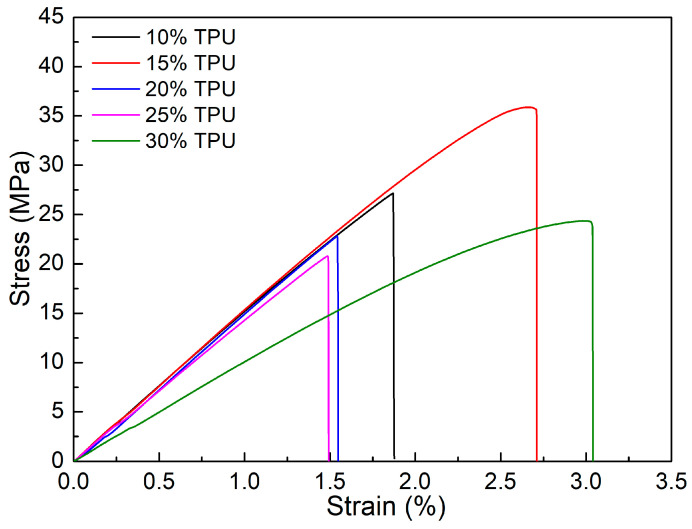
Stress-strain diagrams of ABS/TPU samples at different TPU percentages.

Figure 8 illustrates the stress-strain diagrams of ABS/TPU samples with varying TPU percentages. Blends containing 15 wt% TPU have the highest UTS value, and other blends have comparable UTS values. Like the PP/TPU blends in the previous section, the decline of the UTS value is the low strength of TPU. Blends with 30 wt% TPU have the highest elongation value. Notably, compared with samples without a weld line, the poor weld line bonding reduces the ductility of the blends [13]. The average elastic modulus values of the samples are 15.7 ± 0.9 GPa, 15.5 ± 0.8 GPa, 11.2 ± 0.6 GPa, 14.5 ± 0.8 GPa, and 10.5 ± 0.6 GPa, corresponding to 10 wt%, 15 wt%, 20 wt%, 25 wt%, and 30 wt% TPU percentages. The 10 wt% TPU sample achieves the highest elastic modulus, while the sample with 30 wt% gains the lowest. A representative with 25 wt% TPU with a high elastic modulus value of 14.5 ± 0.8 GPa might be due to the suitable compatibility between TPU and ABS. Generally, increasing the TPU ratio mainly decreases the elastic modulus.

**Figure 9:**
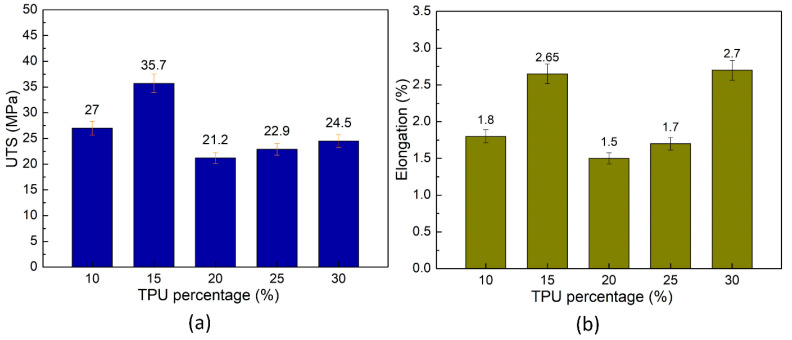
Average mechanical properties of ABS/TPU samples at different TPU percentages: (**a**) average tensile strength and (**b**) average elongation.

Figure 9 presents the average tensile strength and average elongation at the break of ABS/TPU samples at different TPU percentages. The UTS values are 27 ± 1.2 MPa, 35.7 ± 1.8 MPa, 21.2 ± 1.2 MPa, 22.9 ± 1.2 MPa, and 24.5 ± 1.4 MPa, which correspond to TPU samples of 10 wt%, 15 wt%, 20 wt%, 25 wt%, and 30 wt%. From 10–15 wt%, increasing the TPU content leads to higher UTS and elongation values due to good compatibility. On the contrary, adding TPU content from 15–30 wt% results in lower UTS and elongation values due to the lower compatibility rate between TPU and ABS.

A sample containing 15 wt% TPU achieves the highest UTS value of 35.7 ± 1.8 MPa, significantly higher than the other cases, indicating good ABS–TPU compatibility. Meanwhile, the 20 wt% TPU sample has the lowest UTS value of 21.2 ± 1.2 MPa, meaning a low compatibility rate. Remarkably, ABS/TPU blends gain a higher UTS value than PP/TPU blends, as shown in Figure 4 and Figure 9. Moreover, the 15 wt% TPU blends can achieve higher strength and elongation values thanks to new supramolecular interactions between ABS and TPU, which improves the adhesive properties around the weld line [26]. In addition, the elongation-changing pattern is mainly consistent with the UTS value. The high elongation of 2.7 ± 0.2% of the 30 wt% TPU sample could be attributed to the TPU polymer’s ductility. However, compared to samples without weld lines, the ductility values of these samples are lower [13].

**Figure 10:**
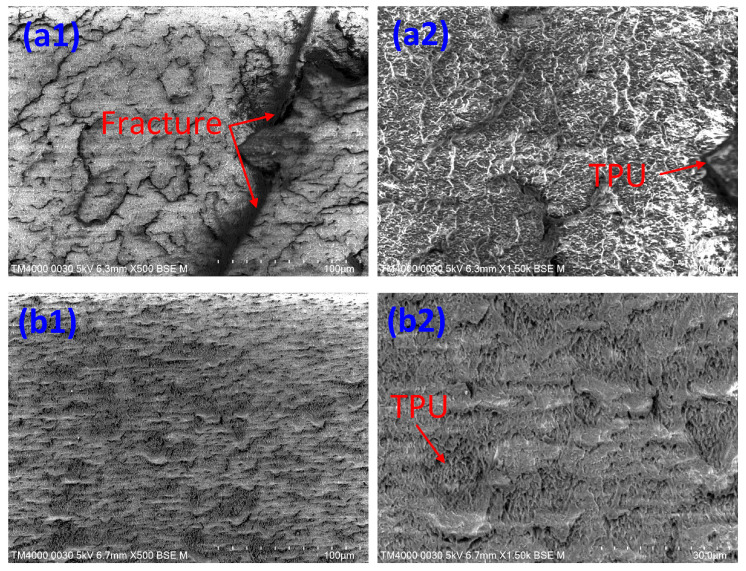
SEM picture of the fracture surface of ABS/TPU samples with 10 wt% TPU and 30 wt% TPU: (**a**) 10 wt% TPU, (**b**) 30 wt% TPU.

Two groups of 10 wt% TPU and 30 wt% TPU are chosen to investigate the fracture surface microstructure. Figure 10 shows the SEM picture of the fracture surface of ABS/TPU samples. Figure 10(a1),(a2) show the SEM picture of the 10% TPU sample at a magnification of 500× and 1500×, respectively. Figure 10(b1),(b2) show the SEM picture of the 30% TPU sample at a magnification of 500× and 1500×, respectively. The dimples in the ABS matrix represent the TPU phase. Because TPU has a higher elongation value, it leaves deeper holes or dimples on the surface after breaking. The 30 wt% TPU sample has a higher rate of dimple area than the 10 wt% TPU sample. In other words, the 30 wt% TPU sample has a rougher surface than the 10 wt% TPU sample, indicating a higher ductility, a consistent result to the De León et al. report [26]. Furthermore, the TPU phase in the 30 wt% TPU sample is more uniformly distributed than in the 10 wt% TPU sample. Different from the PP/TPU rough surface, the smooth surface of the ABS/TPU sample points out a better compatibility mechanism, as shown in Figure 2a.

## 4. Conclusions

This study has investigated the impacts of TPU on the weld line characteristics of the PP/TPU, and ABS/TPU blend polymers. Some remarkable findings could be mentioned:

– In PP/TPU blends, increasing TPU polymer mostly leads to a significant decline in the UTS and elongation values of the PP/TPU composite. Blends with 10 wt%, 15 wt%, and 20 wt% TPU and pure PP gain higher UTS values than blends with recycled PP. The blend with 10 wt% TPU and pure PP achieves the highest UTS value of 21.85 MPa. While the blend with recycled PP achieves a UTS value of 14.4 MPa, the reason is the pure PP’s higher strength than recycled PP. Moreover, with 20–30 wt% TPU, mixing with 25–50 wt% recycled PP mostly leads to higher elongation values than in other cases. SEM results show that PP and TPU are distinct phases; the PP matrix surrounds the TPU phase. As a significantly higher elongation value, the TPU area mainly appears dimly on the fracture surface. Taguchi’s analysis indicates that the TPU factor has a more substantial impact on the mechanical characteristic of the PP/TPU blends than the recycled PP factor.
– In ABS/TPU blends, the sample with 15 wt% TPU achieves the highest UTS value of 35.7 MPa, indicating good compatibility between ABS and TPU. At the same time, the sample with 20 wt% TPU has the lowest UTS value of 21.2 MPa. Additionally, the elongation-changing pattern is most consistent with the UTS value. Notably, SEM results show that the fracture surface of the ABS/TPU blends is flatter than the PP/TPU blend due to a higher compatibility rate. The sample with 30 wt% TPU presents a higher rate of dimple area than the sample with 10 wt% TPU. The TPU phase in the 30 wt% TPU sample is more uniformly distributed than the 10 wt% TPU. Notably, ABS/TPU blends gain a higher UTS value than PP/TPU blends. Increasing the TPU ratio mainly decreases the elastic modulus of both ABS/TPU blends and PP/TPU blends. Both ABS/TPU blends and PP/TPU blends especially experience a decrease in the elastic modulus due to the increasing TPU ratio. The poor bonding in the weld line area results in a drop in the elongation of the blend.

## Figures and Tables

**Table 1:** Molding conditions of the testing samples.

Molding Parameters	Unit	Value for PP/TPU Blend	Value For ABS/TPU Blend
Melt temperature	°C	220	220
Injection pressure	MPa	35	65
Injection time	s	2	2
Drying time (85 °C)	hour	6	12
Holding time to avoid shrinkage	s	0.5	0.8
Holding pressure	MPa	30	50
Injection speed	mm/s	40	35
Cooling time	s	20	20

**Table 2:** Composition of 20 cases of PP/TPU blends.

Group	Case	Pure PP (wt%)	Recycled PP (wt%)	TPU (wt%)
Group 1: 100 wt% pure PP—0 wt% recycled PP	1	90	0	10
	2	85	0	15
	3	80	0	20
	4	75	0	25
	5	70	0	30
Group 2: 75 wt% pure PP—25 wt% recycled PP	6	67.5	22.5	10
	7	63.75	21.25	15
	8	60	20	20
	9	56.25	18.75	25
	10	52.5	17.5	30
Group 3: 50 wt% pure PP—50 wt% recycled PP	11	45	45	10
	12	42.5	42.5	15
	13	40	40	20
	14	37.5	37.5	25
	15	35	35	30
Group 4: 25 wt% pure PP—75 wt% recycled PP	16	22.5	67.5	10
	17	21.25	63.75	15
	18	20	60	20
	19	18.75	56.25	25
	20	17.5	52.5	30
Group 5: 100 wt% recycle PP—0 wt% pure PP	21	0	90	10
	22	0	85	15
	23	0	80	20
	24	0	75	25
	25	0	70	30

## Data Availability

The data used to support the findings of this study are available from the corresponding author upon request.
